# Artificial Intelligence in Rheumatology: Clinical Applications in Rheumatoid Arthritis, Osteoarthritis, and Systemic Lupus Erythematosus

**DOI:** 10.7759/cureus.99108

**Published:** 2025-12-13

**Authors:** Khaled Aldhuaina, Devanshu Gupta, Umbar Bashir, Lathifa Mady Nnap, Akash Rawat, Jelees Dolphin, Razia Sultana, Long Yin Cai, Bashir Imam, Ravi Raj Devkota, Danielle Dsouza, Manju Rai

**Affiliations:** 1 Internal Medicine, Faculty of Medicine, Kuwait University, Kuwait City, KWT; 2 Internal Medicine, Queen's University Belfast, Belfast, GBR; 3 Internal Medicine, Saint James School of Medicine, Park Ridge, USA; 4 Internal Medicine, Faculty of Health Sciences, University of Buea, Buea, CMR; 5 General Medicine, Himalayan Institute of Medical Sciences, Swami Rama Himalayan University, Dehradun, IND; 6 Internal Medicine, University of the West Indies, Kingston, JAM; 7 Internal Medicine, Anwer Khan Modern Medical College, Dhaka, BGD; 8 Internal Medicine, Caribbean Medical University, Willemstad, CUW; 9 Pediatrics, Hurley Medical Center, Michigan State University, Flint, USA; 10 Internal Medicine, China Medical University, Shenyang, CHN; 11 Internal Medicine, DY Patil School of Medicine, Navi Mumbai, Navi Mumbai, IND; 12 Biotechnology, Shri Venkateshwara University, Gajraula, IND

**Keywords:** artificial intelligence, biomarkers, diagnostic imaging, electronic health records, machine learning, osteoarthritis, precision medicine, predictive value of tests, rheumatoid arthritis, systemic lupus erythematosus

## Abstract

Artificial intelligence (AI) has emerged as a transformative force in rheumatology, offering novel diagnostic, predictive, and therapeutic capabilities across chronic inflammatory and autoimmune diseases. This narrative review specifically focuses on rheumatoid arthritis (RA), osteoarthritis (OA), and systemic lupus erythematosus (SLE), where AI applications have been most extensively studied and show the greatest clinical translational potential. In RA, AI applications span early diagnosis via imaging-based models, identification of novel biomarkers through multi-omics integration, and prediction of disease progression and therapeutic response using deep learning algorithms. For OA, AI enhances radiographic interpretation, develops personalized risk prediction models, and enables individualized rehabilitation through wearable and biomechanical data analysis. In SLE, AI aids in biomarker discovery, disease activity monitoring via biosensors, and flare prediction using federated machine learning, with promising applications in high-risk groups. Despite these advances, challenges persist regarding data quality, algorithmic bias, limited explainability, and lack of real-world validation. Ethical considerations surrounding data privacy and equitable access must be addressed to ensure responsible deployment. The review underscores the importance of hybrid human-AI collaboration, integration into electronic health records, and interdisciplinary cooperation to unlock AI’s full clinical potential. Moving forward, research must prioritize transparency, regulatory standardization, and equitable implementation to enhance personalized care in rheumatology. This review consolidates current evidence, highlights key innovations, and identifies future directions essential for advancing AI-driven rheumatologic care.

## Introduction and background

Rheumatic diseases such as rheumatoid arthritis (RA), osteoarthritis (OA), and systemic lupus erythematosus (SLE) are among the most prevalent causes of disability worldwide, with a profound impact on quality of life and healthcare systems [1-3]. These conditions share several clinical challenges: complex and heterogeneous presentations, overlapping symptoms with other disorders, difficulties in achieving early and accurate diagnosis, and substantial variability in treatment response [4-6]. Conventional diagnostic methods, including clinical scoring systems, radiographic imaging, and serological markers, often fail to capture the underlying biological heterogeneity, leading to delayed interventions and suboptimal patient outcomes [7-9].

A data-driven approach offers an opportunity to overcome these barriers. By systematically analyzing multimodal datasets, spanning clinical, imaging, laboratory, genomic, and real-world health records, data-driven models can identify hidden patterns, stratify patients based on risk, predict treatment response, and monitor disease progression [10-12]. Such approaches move beyond traditional “one-size-fits-all” strategies, enabling more precise and personalized rheumatology care. Importantly, this paradigm addresses not only diagnostic accuracy but also the unmet need for individualized therapeutic planning [13,14].

Artificial intelligence (AI), encompassing machine learning (ML) and deep learning (DL) methods, provides the computational backbone for this transformation. AI algorithms have demonstrated success across multiple domains in rheumatology. In RA, AI has been applied to radiographs and MRI for automated joint scoring, to multi-omics datasets for biomarker discovery, and to clinical records for predicting treatment response [15-17]. In OA, DL models have shown promise in detecting subtle radiographic changes, grading severity using the Kellgren-Lawrence (KL) scale, and supporting rehabilitation planning using wearable biomechanical data [18-20]. In SLE, AI has been used to integrate transcriptomic and serological data, forecast flares with biosensor-derived features, and apply federated learning to nephritis risk modeling [21-23]. Collectively, these advances highlight the potential of AI to enhance diagnostic precision, reduce inter-observer variability, and support timely, individualized decision-making in rheumatology.

However, despite this promise, AI applications in rheumatology remain at varying stages of maturity. Many models are limited to proof-of-concept studies, with small or homogeneous cohorts and insufficient external validation. Issues such as algorithmic bias, lack of explainability, and data privacy concerns further hinder clinical integration [24-26]. At present, only a small subset of imaging-based AI tools appears close to clinical adoption, whereas omics-based and wearable-driven models remain largely experimental. Importantly, most existing studies are retrospective, single-centre, and pre-deployment, underscoring that the current evidence base should be regarded as early-stage rather than reflective of mature, clinically integrated technologies.

While several prior reviews have summarized AI applications in rheumatology, most have either focused on individual diseases or emphasized technical aspects without integrating clinical translation. Our manuscript adds to this body of work by providing a comparative, disease-specific synthesis across RA, OA, and SLE, critically appraising study quality, dataset characteristics, and clinical readiness. By incorporating structured summary tables and directly contrasting AI approaches with conventional diagnostic and therapeutic methods, this review highlights not only innovations but also persisting gaps in real-world validation, regulatory pathways, and equity of access. Unlike prior reviews that typically examine AI applications within a single rheumatic disease or focus primarily on technical model development, this review adopts a tri-disease comparative framework across RA, OA, and SLE, enabling cross-condition insights into diagnostic, prognostic, and translational readiness. By integrating evidence across these three major conditions, the review provides a unified synthesis of advances in AI-driven diagnosis, disease activity assessment, prognostic modeling, and personalized therapeutic approaches.

## Review

Methodology

Given the rapidly evolving landscape of AI in rheumatology, we undertook a structured narrative review to consolidate current evidence. Although not a systematic review, we adopted measures to enhance transparency, reproducibility, and reliability. An extensive literature search was conducted in PubMed, Cochrane Library, Scopus, and Web of Science between January 1, 2013, and October 25, 2025, covering the past decade to reflect the most relevant AI applications in healthcare. The following Medical Subject Headings (MeSH) terms and keywords were used in combination with Boolean operators: “Artificial Intelligence,” “Machine Learning,” “Deep Learning,” “Rheumatology,” “Rheumatoid Arthritis,” “Osteoarthritis,” “Systemic Lupus Erythematosus,” “Biomarkers,” “Predictive Models,” and “Precision Medicine.”

We included peer-reviewed original articles, reviews, meta-analyses, and conference proceedings in English that reported on the application of AI to diagnosis, prognosis, monitoring, or treatment of RA, OA, or SLE. Both human and translational studies were eligible. Studies were excluded if they did not clearly describe AI methodology, lacked clinical or translational relevance, or were limited to theoretical models without validation.

Two reviewers independently screened titles and abstracts, followed by a full-text review to minimize selection bias. Disagreements were resolved through consensus. Reference lists of key studies were also hand-searched to identify additional relevant articles. Although formal risk-of-bias (RoB) tools (e.g., Cochrane RoB) were not applied due to the heterogeneity of study designs, we performed a qualitative assessment focused on broad indicators such as dataset size, validation approach, and general methodological transparency; however, we acknowledge that this appraisal does not constitute a structured or graded RoB evaluation. Priority was given to studies with external validation, multicenter datasets, or integration with real-world clinical practice.

**Figure 1:**
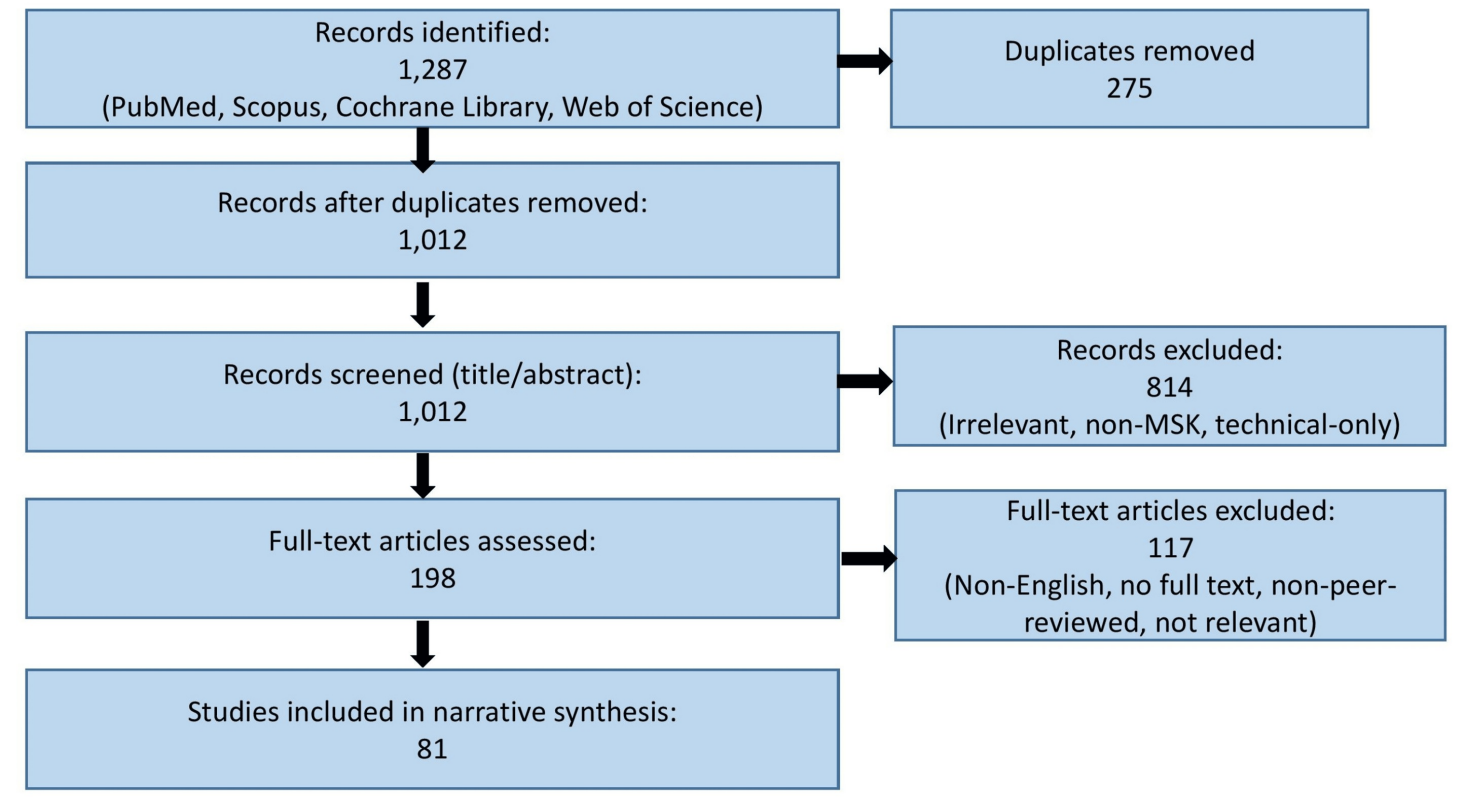
Flow diagram illustrating the study identification, screening, eligibility assessment, and inclusion process. MSK: musculoskeletal

By adopting these structured methods, we aimed to enhance the reliability and transparency of this narrative review while acknowledging the inherent limitations compared with systematic approaches. The study selection process is summarized in a Preferred Reporting Items for Systematic Reviews and Meta-Analyses (PRISMA)-style flow diagram (Figure 1).

Applications of AI in rheumatology

AI is increasingly transforming the landscape of rheumatology by offering advanced tools to support clinical decision-making, improve diagnostic accuracy, personalize treatment strategies, and enhance patient monitoring. Given the complexity and overlapping manifestations of rheumatic diseases, AI can assist clinicians by analyzing large volumes of clinical, imaging, and molecular data to provide early and precise diagnostic insights [17]. ML and DL models are capable of detecting subtle patterns in data, enabling earlier disease identification and more targeted therapeutic interventions [18].

Beyond diagnosis, AI is being used to develop prognostic models that predict disease progression, flares, and therapeutic responses, thereby facilitating individualized care planning. Natural language processing (NLP) further enables efficient extraction of critical information from unstructured clinical documentation, improving workflow and data utilization. Additionally, AI-powered tools are being incorporated into telemedicine platforms, allowing for remote patient monitoring through imaging interpretation, wearable technology, and mobile applications [19].

Several AI-based systems, including image analysis tools for joint assessments and clinical decision support algorithms, are already under investigation or in preliminary use. These developments highlight AI's growing role in advancing precision medicine within rheumatology, addressing current gaps in diagnosis, prognosis, and long-term disease management. Despite major advances, significant diagnostic gaps persist across rheumatic diseases, including delayed recognition of early or atypical presentations, limited sensitivity of conventional biomarkers, and substantial inter-observer variability in imaging interpretation. These challenges are further compounded by disease heterogeneity, which often leads to missed or late diagnoses and underscores the need for more precise, data-driven diagnostic tools.

AI in early diagnosis and management of RA

Early detection of RA is critical for initiating appropriate therapy and potentially preventing long-term joint damage. AI is being increasingly explored for this purpose, leveraging its capacity to analyze diverse data types with high precision.

Imaging Analysis

DL algorithms, particularly convolutional neural networks (CNNs), have demonstrated impressive accuracy in interpreting digital X-ray images of hands and feet. These models can detect subtle changes such as joint erosions and space narrowing, sometimes with performance comparable to expert radiologists. In one study of 670 participants, including 291 confirmed RA patients, 223 individuals with other autoimmune conditions, and 156 healthy controls, an AI-powered artificial neural network (ANN) surpassed rheumatologists in diagnostic accuracy, achieving 90.6% accuracy [20]. However, this study was retrospective, involved a relatively small sample size, and did not undergo independent external validation, limiting its generalizability across diverse populations.

AI tools analyzing MRI scans can detect early inflammatory markers like synovitis and bone marrow edema, often before they are visible on X-rays. Nonetheless, these models were benchmarked against surrogate scoring systems such as the Rheumatoid Arthritis Magnetic Resonance Imaging Score (RAMRIS) rather than prospective clinical outcomes, and their diagnostic accuracy varied by anatomical region, underscoring the need for broader validation.

AI algorithms applied to ultrasound imaging have also shown promise in detecting early joint inflammation. A 2024 study reported high diagnostic performance, with area under the curve (AUC) values ≥0.90 for detecting synovitis across multiple joints [24]. However, these models were trained on expert-annotated datasets, introducing subjectivity, and were evaluated in relatively small patient cohorts (<200), which limits extrapolation to broader populations.

Beyond conventional CNN-based image interpretation, novel approaches are integrating robotics with AI. A recent study evaluating the automated robotic ultrasound system ARTHUR V.2.0 combined with the AI model DIANA V.2.0 in 30 RA patients achieved an 85.5% scanning success rate, with repeatability comparable to intra-expert agreement [25]. The system demonstrated substantial concordance with expert rheumatologist and independent assessor evaluations for synovial hypertrophy and Doppler activity, underscoring its potential for standardized and automated joint assessment, although further refinement is required to optimize accuracy across specific joints.

Biomarker Identification and Data Integration

AI-driven analyses of high-dimensional omics data, including transcriptomic, proteomic, and epigenetic datasets, have substantially advanced biomarker discovery in RA. ML techniques such as the Least Absolute Shrinkage and Selection Operator (LASSO) have been used to identify platelet-derived biomarkers like transforming growth factor-β (TGF-β) and its downstream effector mitogen-activated protein kinase 3 (MAPK3), both of which are implicated in RA pathogenesis [26].

These models are also capable of stratifying RA patients by serological status and distinguishing them from healthy individuals, based on combinations of markers like rheumatoid factor (RF), anti-citrullinated protein antibodies (ACPA), C-reactive protein (CRP), erythrocyte sedimentation rate (ESR), and clinical symptoms. This data-driven approach enhances diagnostic precision, especially in undifferentiated arthritis cases [27].

AI in Therapy Response Prediction

Optimizing RA treatment through personalized approaches is another frontier where AI is making significant strides. One of the most transformative applications of AI in RA is its capacity to predict therapeutic response, fostering a shift toward precision medicine. ML models can analyze baseline demographic and clinical variables alongside serological and molecular data to predict responses to conventional synthetic disease-modifying antirheumatic drugs (csDMARDs), biologic agents (bDMARDs), and targeted synthetic DMARDs (tsDMARDs) such as JAK inhibitors [28,29]. Algorithms such as random forests, support vector machines, and neural networks (NNs) are being used to build therapy response models [30].

Imaging-based prediction via AI can uncover inflammation or damage patterns predictive of therapeutic outcomes [29]. Explainable AI (XAI) tools like Shapley value analysis enhance model transparency, clarifying how specific clinical inputs influence therapy response predictions, thereby facilitating clinical acceptance [27].

AI in RA Diagnosis and Treatment Response

RA diagnosis has traditionally relied on radiographic scoring systems, such as the Sharp/van der Heijde (SvdH) score, and on the detection of serological and molecular biomarkers [31,32]. While these tools assess disease severity, particularly features like joint space narrowing (JSN) and erosions, they are inherently subjective and prone to interobserver variability [33].

Prediction Models for Disease Activity and Progression

ML is increasingly being used to forecast RA disease progression by integrating clinical parameters, imaging features, and biomarker profiles. These models facilitate early identification of aggressive disease phenotypes, allowing timely therapeutic intervention. Guan et al. developed a Gaussian process regression model using demographic, clinical, and genetic data from the DREAM RA Responder Challenge cohort (n=1,892 training; n=680 testing) to predict responses to tumor necrosis factor-alpha (TNF-α) inhibitors. The model achieved ~78% classification accuracy with correlation coefficients of 0.405 (training) and 0.393 (testing), while maintaining clinical interpretability and usability in routine practice, even by non-expert users [34].

Furthermore, DL models trained on longitudinal datasets have shown promise in predicting disease flares and sustained remission. The AI-powered Rheuma Care Manager (RCM) demonstrated good predictive performance for RA flare risk (sensitivity: 72%, specificity: 76%, area under the receiver operating characteristic curve (AUROC): 0.80) [29]. Access to the tool improved physicians’ decision confidence (from 3.5/5 to 3.7/5), reduced variability in flare risk assessments (deviation reduced from 20% to 12%), and increased the rate of treatment reductions (from 42% to 50% vs. 20% without the tool). Usability was rated as good (System Usability Scale (SUS): 82/100) with high acceptance (Net Promoter Score (NPS): 7/10), suggesting that RCM could effectively support consistent and confident treat-to-target decisions in clinical practice. These tools integrate diverse datasets to stratify patients based on projected disease trajectory, enabling more personalized treatment planning.

Predicting Therapeutic Response and Personalizing Treatment

Tao et al. demonstrated the utility of ML in differentiating responders to TNF-α inhibitors. Adalimumab responders showed upregulated TNF signaling pathways, while etanercept responders exhibited unique hypermethylation signatures, despite having similar clinical characteristics such as age and RF status [35]. This highlights the limitation of relying solely on clinical features and emphasizes the value of molecular profiling. Gosselt et al. further showed that ML approaches like LASSO could outperform traditional logistic regression (LR) by evaluating a broader spectrum of biomarkers when predicting methotrexate response, particularly in heterogeneous sample populations [36]. AI also contributes to imaging-based therapeutic response prediction by identifying patterns of inflammation or structural damage that correlate with treatment efficacy [37].

AI in OA care

AI in Radiographic Interpretation

X-rays and MRIs serve as the primary imaging modalities for diagnosing OA, commonly assessed using the KL grading system [30]. Despite their widespread use, these techniques are inherently subjective and often suffer from limited inter-observer reliability, underlining the necessity for AI-based tools to automate evaluation and improve diagnostic precision [38-40].

Several AI-driven approaches for OA diagnosis have shown encouraging results. In a diagnostic accuracy study conducted by Mourad et al., 276 patients (mean age 46.7 ± 16.4 years; range 18-75; male-to-female ratio 1.86:1) with 397 cone beam computed tomography (CBCT) records were evaluated using a You Only Look Once (YOLO)-based NN for radiographic confirmation of temporomandibular joint osteoarthritis (TMJ-OA) [41]. The model showed substantial to near-perfect agreement with expert examiners, with AUC values ranging from 0.872 (surface erosion) to 0.911 (subcortical cyst), supporting its potential as a valid and automated diagnostic tool. Despite these encouraging results, the study was cross-sectional, limited to TMJ OA, and did not assess longitudinal progression or external clinical applicability. Tiwari et al. analyzed 2,068 knee radiographs from adults with OA, using eight transfer-learning DL models and benchmarking their performance against KL grades assigned by three experienced orthopedic surgeons [42]. DenseNet201 achieved the highest accuracy (92.9% vs. trainee 74%), outperforming ResNet50, VGG-16, InceptionV3, MobileNetV2, EfficientNetB7, Xception, and NasNetMobile, highlighting its promise for automated orthopedic radiograph classification and differentiation of symptomatic versus asymptomatic OA.

Although advancements in AI-assisted OA diagnosis are notable, many models have yet to be validated in real-world clinical environments. Tandon et al. assessed GPT-4o and reported high sensitivity (0.886) and recall (0.95), indicating strong performance, albeit with a notable rate of false positives, suggesting the need for caution in imaging applications [43]. Similarly, Temel et al. found that its sensitivity and accuracy were consistently low across all KL grades, thereby limiting its utility in grading and distinguishing OA severity [44].

Risk Assessment Models

Effective risk assessment tools are crucial for the early prevention and intervention of OA. Current methods are often inadequate, as they tend to depend on clinical symptoms that emerge only during advanced stages of the disease, thereby delaying appropriate treatment [44]. This underscores the need for ML models that integrate diverse variables to more accurately predict OA risk.

Recent developments in ML have introduced innovative techniques for OA risk prediction. Fu et al. evaluated five ML algorithms and found that while LR, light gradient boosting machine (LGBM), random forest, and XGBoost each demonstrated moderate performance with AUC values ranging from 0.7715 to 0.7888, the CatBoost algorithm achieved the highest AUC of 0.802 [45]. This suggests that CatBoost may be particularly effective in identifying individuals at elevated risk for OA and could be a valuable tool in clinical decision-making. However, this model was trained on a limited age group (≥45 years) and may not generalize to younger populations with different risk profiles. Additionally, a model developed using the Tree-based Pipeline Optimization Tool showed potential in predicting risk factors for contralateral knee OA, though its performance was modest (AUC-ROC 0.58), indicating the need for further refinement [46].

Lazzarini et al. (2021) developed a comprehensive risk prediction tool integrating electronic health records, genetic markers, and lifestyle factors, demonstrating improved sensitivity for identifying high-risk individuals in primary care settings [47]. Thomas et al. (2022) introduced novel DL architectures for analyzing MRI data, achieving remarkable accuracy in predicting cartilage loss progression with potential for early intervention strategies [48]. McCabe et al. (2022) conducted multi-center validation studies confirming the generalizability of AI risk models across diverse populations, while addressing important considerations regarding algorithmic bias and healthcare equity [49]. These developments indicate that while AI-based risk assessment tools show promising potential, they remain in the early stages of validation and are not yet ready for clinical deployment, highlighting the need for more rigorous testing before they can meaningfully influence OA prevention and management strategies.

Personalized Therapy Development Using AI

AI has revolutionized personalized therapy development for OA by enabling precision medicine approaches that tailor treatment strategies to individual patient characteristics. Recent advances demonstrate the potential of AI models to optimize therapeutic outcomes through patient stratification and treatment response prediction. Twumasi et al. tested the “Slider” home rehabilitation device on 32 participants, and their bilateral knee kinetic data were analyzed using four unsupervised clustering techniques to compare performance and identify predictive factors [50]. The analysis revealed three distinct, time-varying movement patterns for each knee, with hierarchical clustering performing best for the right knee and CLARA (Clustering Large Applications) for the left. BMI was the most influential predictor for the right knee, with higher BMI reducing the likelihood of belonging to one movement cluster but increasing the odds of belonging to another, while gender was the strongest determinant for the left knee, with males significantly more likely to be classified in a specific cluster. These insights offer a basis for tailoring rehabilitation protocols, predicting recovery trajectories, and minimizing postoperative complications through more personalized interventions [50]. While innovative, the device was evaluated in a small pilot cohort, and its clinical utility for large-scale personalized OA rehabilitation remains to be validated.

In another effort to personalize OA treatment, Heidari et al. proposed a Quantum Neural Network (QNN) model grounded in precision medicine principles [51]. Their model categorized patients based on therapeutic response and achieved a sensitivity of 0.82, although its specificity was limited to 0.26, indicating it was proficient in identifying true positives but also prone to false positives. Despite its encouraging findings, the study was conducted on a small scale and has not been validated on large, unstructured clinical datasets, limiting its external generalizability and real-world applicability.

Arbeeva et al. developed AI algorithms combining clinical data with genetic markers to personalize pain management strategies, demonstrating significant improvements in treatment efficacy compared to standard care protocols [52]. Recent investigations have expanded into precision drug development. Rockel et al. utilized DL models to identify novel therapeutic targets by analyzing multi-omics data from OA patients, leading to the discovery of personalized combination therapies [53]. Concurrently, Liu et al. introduced reinforcement learning approaches for optimizing exercise therapy prescriptions, adapting rehabilitation protocols based on real-time patient feedback and progression metrics [54].

These developments highlight AI's transformative potential in creating truly personalized OA treatments, moving beyond one-size-fits-all approaches toward precision interventions that maximize therapeutic benefit while minimizing adverse effects for individual patients.

Advancing SLE assessment with AI

AI for Biomarker Discovery

The multi-omics and ML-driven study by Zhou et al. analyzed transcriptomic, single-cell, and metabolomic data to elucidate oxidative stress (OS) mechanisms in SLE [55]. The study identified significant disruptions in OS-related metabolic pathways and highlighted six key genes (ABCB1, AKR1C3, EIF2AK2, IFIH1, NPC1, SCO2) with distinct expression patterns across immune cell subsets, correlating strongly with OS levels, antioxidant status, and key metabolites, thereby offering potential biomarkers and therapeutic targets for early detection and precision management of SLE. Yet, this study was exploratory in nature, with findings requiring replication in larger, ethnically diverse cohorts before clinical adoption. Similarly, He et al. (2025) developed a hybrid DL model that integrates handcrafted biochemical features with deep sequence modeling to improve the prediction of SLE-associated epitopes [56]. The model achieved high predictive performance (ROC AUC = 0.9506, F1-score = 0.8333), outperforming traditional algorithms, and offers biologically interpretable insights that can advance immunotherapeutic design and autoimmune disease research. Nevertheless, the model performance was assessed retrospectively, and the lack of validation in prospective clinical cohorts limits its immediate translational value. The practical application of such AI-driven methodologies is evident in the Accelerating Medicines Partnership (AMP) lupus nephritis (LN) cohort, where elevated levels of anti-C1q, anti-dsDNA, and anti-ribosomal P antibodies were linked to proliferative LN, showcasing the potential of AI-enhanced serological analysis [57].

AI for Disease Activity Monitoring

Remote monitoring technologies, widely adopted in diabetes management through continuous glucose monitoring systems, are now being adapted for use in complex autoimmune conditions like SLE. Advanced biosensors are now being employed to continuously monitor dermal temperature, interleukin concentrations, and heart rate variability in real time. These advanced sensors feed data into patient-facing dashboards that are integrated with clinician platforms, enabling healthcare providers to detect disease flares early and make timely therapeutic decisions [58]. Such systems hold promise for facilitating early intervention in high-risk groups, particularly in populations with added clinical complexity, such as pediatric and geriatric patients.

AI For Risk Stratification

In a study, data from 219 Omani patients, including 138 with SLE and 81 with other rheumatologic conditions, were analyzed using the CatBoost algorithm with recursive feature selection to predict SLE presence, with explanations generated through the SHAP model and validated by rheumatologists [59]. The framework achieved high accuracy (AUC = 0.95, sensitivity = 92%) and identified alopecia, renal disorders, acute cutaneous lupus, haemolytic anaemia, and age as key predictors, supporting its potential for early diagnosis and timely clinical intervention. However, this study acknowledges the limitation of imbalanced classification, where the underrepresentation of certain cases in the dataset may bias performance evaluation and affect the generalizability of the results. Similarly, in this retrospective study of 780 patients with LN at West China Hospital, Yang et al. (2024) developed and validated nine ML models using clinical and laboratory data to predict proliferative LN when renal biopsy is not feasible [60]. The random forest model demonstrated the highest diagnostic performance (AUC = 0.880, 95% confidence interval (CI): 0.835-0.926), and key predictors included traditional markers such as anti-dsDNA antibodies, complement levels, and serum creatinine, along with emerging indicators like serum chloride, neutrophil percentage, and serum cystatin C, supporting a reliable, non-invasive diagnostic approach for PLN. This study is limited by its single-center, retrospective design, and the findings have not yet been validated in multicenter cohorts. Therefore, external validation and refinement using data from diverse populations are essential before clinical implementation. Moreover, due to concerns about data completeness, variables with more than 30% missing values were excluded, which may have prevented the models from incorporating other potentially important predictive features. However, these individualized prognostic tools are particularly valuable for high-risk groups, including infants with congenital lupus, patients with diabetes, and elderly individuals presenting with atypical SLE.

Despite the promise of AI technologies such as federated learning and biosensor-based monitoring in managing SLE, their readiness for routine clinical use demands critical evaluation. While federated learning offers advantages in data privacy by allowing models to be trained across decentralized data sources, their generalizability across diverse patient populations remains uncertain without thorough external validation. Additionally, integrating multi-omics datasets with electronic medical records (EMRs) poses significant challenges due to technical limitations and the high computational load.

Further, advances in histological image classification using 20-plex high-resolution immunohistochemistry combined with AI have enabled detailed identification of immune cell populations in renal biopsies, offering insights into the pathophysiology of LN and supporting targeted therapeutic strategies [61]. Overall, AI systems are beginning to reshape research and early diagnostic pathways, from biomarker identification and disease monitoring to predicting complications. Future research should focus on embedding these technologies into clinical practice while ensuring equitable access for diverse patient groups.

To provide a structured overview, Table 1 summarizes key studies applying AI across RA, OA, and SLE, highlighting their methodologies, datasets, clinical applications, findings, and limitations.

**Table 1:** Applications of artificial intelligence in rheumatology. RA: rheumatoid arthritis, OA: osteoarthritis, SLE: systemic lupus erythematosus, ANN: artificial neural network, ML: machine learning, XGBoost: extreme gradient boosting, DLM: deep learning model, KL: Kellgren-Lawrence, SHAP: Shapley additive explanations, LN: lupus nephritis, LLM: large language model; TNF: tumor necrosis factor

Disease	Author/Year	AI Method/Model	Sample Size	Validation Type	Clinical Application	Main Limitation
RA	Bai et al., 2022 [20]	ANN	N=670 (291 RA, 223 autoimmune, 156 healthy controls)	Internal validation	Diagnosis via radiographs	Retrospective, limited sample diversity; no external validation
RA	Liu et al., 2023 [26]	Multi-omics AI integration	N=440 (344 RA, 96 healthy controls)	None	Biomarker discovery	No external validation; small sample; limited clinical covariates analyzed
RA	Kim et al., 2024 [27]	ML (XGBoost integrating clinical + serology)	N=783 (574 anti-TNF, 209 anti-JAKi therapy)	Internal validation	Early RA diagnosis and treatment response	Limited generalizability; no external validation across diverse cohorts
OA	Tiwari et al., 2022 [42]	8 transfer learning DLMs (DenseNet201 best)	N=2068 radiographs	Internal validation	KL grading of knee OA	Single-center dataset; external validation lacking; performance drop with heterogeneous images
OA	Tandon et al., 2025 [43]	LLM (GPT-4o) for OA vs. normal classification	N=1000 X-rays (500 OA, 500 normal)	External validation	Diagnosis and risk prediction	Low specificity; limited suitability as a standalone diagnostic tool
OA	Temel et al., 2025 [44]	LLM (GPT-4o) as decision support	N=226 knee OA X-rays	External validation	KL grading	Very low accuracy and sensitivity; poor grade differentiation
SLE	Zhou et al., 2025 [55]	Deep learning (multi-omics)	N=1493 (1355 SLE, 138 healthy controls)	None	Biomarker discovery	Heterogeneity; no external validation
SLE	AlShareedah et al., 2023 [59]	CatBoost + SHAP	N=219 (138 SLE, 81 other rheumatic diseases)	Internal validation	Prognosis/early prediction	Single-center; class imbalance; limited external generalizability
SLE	Yang et al.,2024 [60]	Conventional ML	N=780 (547 training, 233 testing)	Internal validation	Proliferative LN risk prediction	No multicenter or external validation; uncertain generalizability
SLE	Louis Sam Titus et al., 2023 [61]	AI histological classification	N=18 LN patients (two centers)	Internal validation	Pathophysiology mapping	Very small sample; early-stage method; no external validation

Challenges and limitations

Data Quality and Diversity

The effectiveness of AI models in rheumatology relies heavily on the quality and inclusiveness of the datasets used. However, research by Al Zo’ubi [62] and Purohit et al. [63] indicates that current data sources are frequently fragmented, regionally biased, and often fail to include rare rheumatologic conditions. Chandwar and Misra [64] further highlight challenges such as inconsistent data labeling and the lack of interoperability among EHR systems, which undermine the generalizability and reliability of AI tools across diverse patient populations. As a result, these models may perform poorly for underrepresented groups, contributing to disparities in healthcare delivery.

Ethical and Regulatory Concerns

Systemic bias present in retrospective datasets can be inadvertently reinforced by AI systems, as noted by Sequí-Sabater and Benavent [65]. Patient privacy is another pressing concern, especially in the context of rapidly advancing digital technologies, as emphasized by Mondillo et al. [66]. Moreover, with increasing reliance on cloud-based infrastructure, Ray stresses the urgent need for robust frameworks that define data ownership and guide ethical AI use [67]. The absence of standardized global regulations further complicates the international deployment of AI in healthcare.

Lack of Explainability in AI Models

A significant barrier to clinical adoption is the opaque nature of many advanced AI models. As Ray [67] and Knitza et al. [68] argue, the inability to understand or explain how these models generate decisions creates distrust among clinicians. In specialties like rheumatology, where clinical judgment is often complex and individualized, the lack of transparency in DL systems hinders meaningful integration into routine care.

Lack of Prospective Clinical Validation

While AI systems show promise in controlled environments, their translation into clinical practice remains inconsistent. Knitza and colleagues, in two multicenter studies [68,69], found variable diagnostic performance of AI-based symptom checkers. This highlights the necessity for robust prospective trials and standardized implementation protocols. Experts such as Chan [70] and AlGhazo et al. [71] emphasize that validation in real-world settings is critical to ensuring the clinical effectiveness and reliability of AI tools in rheumatology.

Overfitting is a notable limitation in AI models, where high performance on training datasets does not always translate to clinical utility. Vodencarevic et al. (2021) highlighted that while some AI systems perform well on curated data, their effectiveness significantly diminishes when applied to clinical scenarios, reducing their value for patient care at scale [72]. This issue was similarly demonstrated by Brown et al. (2024), who developed an AI system using echocardiography to diagnose rheumatic heart disease [73]. They observed reduced sensitivity when using different ultrasound machine brands or in cases of poor image quality. The authors suggested adapting AI algorithms to be more compatible with a variety of devices, particularly handheld systems, to expand diagnostic access in low-resource settings such as rural Uganda.

Another challenge stems from the limitations of specific ML algorithms. For example, Bai et al. (2022) used ANNs to improve the diagnostic accuracy of RA, outperforming traditional threshold-based methods (90.6% vs. 88.8%) [20]. However, the ANN model relied on a small number of features, primarily age and anti-cyclic citrullinated peptide (anti-CCP) levels, which limited its ability to generalize and offer a full clinical picture. Bai et al. proposed combining other ML techniques, such as CNNs, which can incorporate image data like radiographs, to enhance diagnostic breadth and utility.

Although promising, most AI applications in rheumatology remain at a proof-of-concept stage. For RA, CNNs trained to detect joint erosions and synovitis have shown diagnostic accuracy comparable to that of radiologists, but they have not yet been tested in prospective multicenter cohorts. Similarly, in OA, DenseNet201 and ResNet50 demonstrate high accuracy in retrospective datasets but lack validation in longitudinal clinical trials. NLP-based tools using EHRs have been piloted to forecast outcomes in RA, but these models require further refinement before integration into decision-support systems. In SLE, federated ML for nephritis prediction and biosensor-based disease activity monitoring remain largely experimental, with no regulatory approval for routine use.

Taken together, only a small subset of imaging-based AI tools (e.g., radiographic scoring automation for RA and OA) appear close to clinical integration, while omics-driven and wearable-based models require much more robust validation. This distinction highlights the need for prospective multicenter trials, external validation across diverse populations, and demonstration of tangible improvements in clinical decision-making and patient outcomes before widespread adoption is feasible.

To further contextualize AI innovations in rheumatology, it is important to compare them directly with conventional diagnostic and management approaches. Table 2 provides this comparison, outlining the added value and persisting gaps of AI across RA, OA, and SLE.

**Table 2:** Comparison of traditional approaches and AI applications in rheumatology. RA: rheumatoid arthritis, RAMRIS: rheumatoid arthritis magnetic resonance imaging score, CNNs: convolutional neural networks, ML: machine learning, RF: rheumatoid factor, ACPA: anti-citrullinated peptide antibodies, ESR: erythrocyte sedimentation rate, CRP: C-reactive protein, OA: osteoarthritis, KL: Kellgren-Lawrence, EHRs: electronic health records, SLE: systemic lupus erythematosus, anti-dsDNA: anti-double-stranded DNA antibodies, IHC: immunohistochemistry

Disease	Traditional Approach	AI Approach	Added Value of AI	Remaining Gaps
RA	Radiography and manual scoring (RAMRIS, Sharp score)	CNNs for joint erosion/synovitis detection; ML for biomarker integration	Earlier diagnosis, automated quantification, and improved prognostic modeling	Limited generalizability, external validation required
RA	Serological markers (RF, ACPA, ESR, CRP)	Multi-omics AI integration (genomics, proteomics)	Enhanced biomarker discovery and patient stratification	Lack of large, diverse datasets; interpretability issues
OA	Radiographic KL grading by human raters	Deep learning models (DenseNet201, CNNs)	Higher accuracy, reduced inter-observer variability, and outperform trainees	Single-center studies, no regulatory approval yet
OA	Physical exam and generic rehab protocols	AI using wearable/biomechanical data	Personalized rehab, real-time functional monitoring	Limited adoption and integration challenges with EHRs
SLE	Serology (anti-dsDNA, complement levels), clinician scoring	Deep learning for biomarker integration; AI flare prediction models	Improved risk prediction, real-time monitoring, and early flare detection	High heterogeneity, need for multicenter prospective validation
SLE	Renal biopsy manual histology interpretation	AI histopathology (IHC + deep learning)	Finer cellular mapping, discovery of novel immunological pathways	Early-stage, requires clinical translation and validation

Future Directions

The future of AI in rheumatology lies in developing hybrid human-AI collaborative frameworks that enhance clinical decision-making rather than replacing physicians. AI could boost clinicians' knowledge and support their decision-making to create an "artificially enhanced rheumatologist" [74]. A critical advancement will be seamless EHR integration, enabling real-time risk stratification and predictive alerts for disease flares, particularly in complex conditions like SLE [75].

Multi-omics integration represents a transformative frontier, where AI algorithms will synthesize genomic, proteomic, and metabolomic data to enable truly personalized treatment selection in RA and other rheumatic diseases [76,77]. This approach promises to move beyond traditional trial-and-error prescribing toward precision therapeutics based on individual molecular signatures [78].

Federated learning architectures will enable collaborative AI model development across institutions while preserving patient privacy, potentially accelerating research in rare rheumatic conditions [79]. Additionally, continuous learning systems that adapt to real-world clinical outcomes will improve predictive accuracy over time [80].

Critical challenges remain in ensuring algorithmic transparency, mitigating bias, and establishing regulatory frameworks for clinical AI deployment [81]. Comprehensive training programs for rheumatologists in AI literacy and interdisciplinary collaboration between clinicians, data scientists, and ethicists are essential. Success will depend on rigorous validation studies, patient-centered design, and commitment to health equity in AI tool development.

## Conclusions

Early evidence suggests that AI holds transformative potential in rheumatology, offering novel tools for early diagnosis, risk stratification, and personalized treatment of complex diseases such as RA, OA, and SLE. By integrating multimodal data, ranging from imaging and omics to electronic health records, AI enables more precise and individualized care pathways. Its application in predictive modeling, therapeutic response forecasting, and remote patient monitoring has already demonstrated promising results. However, significant barriers remain, including data quality issues, lack of explainability, ethical concerns, and limited real-world validation. To unlock AI’s full potential, collaborative frameworks that combine human expertise with machine intelligence must be developed and rigorously tested. Future efforts should focus on enhancing model transparency, integrating AI seamlessly into clinical workflows, and ensuring equitable access to AI-driven innovations. With sustained interdisciplinary collaboration and ethical vigilance, AI can significantly advance the future of rheumatologic care.
